# Ian Reid MB, ChB, PhD, FRCPsych: Formerly Professor of Mental Health and Head of Department, School of Medicine and Dentistry, University of Aberdeen

**DOI:** 10.1192/pb.bp.116.053777

**Published:** 2016-10

**Authors:** Ross Hamilton, Alastair Palin

**Figure:**
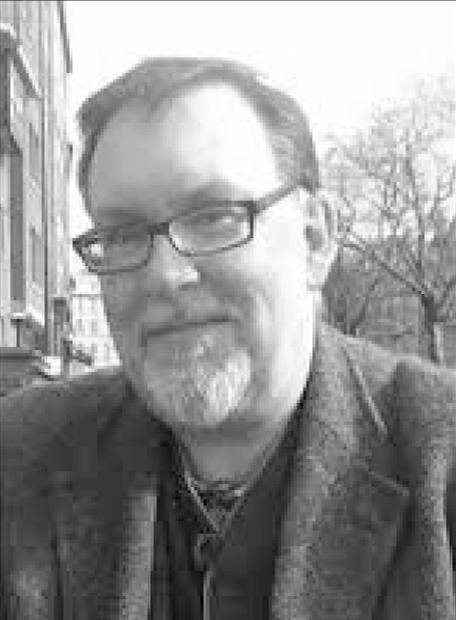


Ian Reid, who died tragically early at the age of 53 on 15 June 2014, played a leading role in British, especially Scottish academic psychiatry for over two decades. His particular interest was in electroconvulsive therapy (ECT), which he regarded as a neglected, undervalued but highly effective form of treatment for depression and bipolar illnesses. He was passionate in his attempts to destigmatise ECT and to puncture the myths surrounding this controversial treatment, while highlighting that it was safe, effective and evidence based. He made successful bids to extend the work of his research team into the psychometrics of mood and at the time of his death, he and his team – through collaboration with the neuroimaging team in Aberdeen – were discovering exciting new biomarkers for mood disorder and the mode of action of ECT. Ian was a founding member of the Scottish ECT Accreditation Network and became Chair of the Royal College of Psychiatrists Special Committee on ECT and Related Treatments in 2011.

He was also an effective advocate for the appropriate use of antidepressant medication. His collaborative research with primary care colleagues in Aberdeen into prescribing of anti-depressant medication led directly to the withdrawal of a Scottish Government Health Efficiency Access Treatment target to reduce antidepressant prescribing. This research, quoted by the Public Audit Committee of the Scottish Government, had widespread media coverage and led to a robust debate in the *BMJ*.

Internationally, he collaborated, for example, with colleagues in India, via the Trusted Mobile Platform for the Self-Management of Chronic Illness in Rural Areas.

He was a prolific contributor to the literature, editing and contributing chapters to the standard textbook on the subject, *Fundamentals of Clinical Psychopharmacology*, as well as being associate editor of *Therapeutic Advances in Psychopharmacology*. He regularly reviewed papers for numerous other journals. Ian was a long-standing member and staunch supporter of the British Association for Psychopharmacology (BAP) and an elected member of BAP Council for 4 years. He contributed much to the BAP over the years, including teaching at educational events and supporting young colleagues presenting research findings at the annual meetings.

Highly active on the Scottish psychiatry scene, Ian was an expert panel member of the Scottish Medicines Consortium and of the Scottish Intercollegiate Guidelines Network review group. He was also a member of the Healthcare Improvement Scotland Integrated Care Pathways for Mental Health steering group, which set national standards for healthcare in Scotland, and chaired their bipolar disorder subgroup.

Despite his national and international responsibilities, he kept his feet very much on the ground in Scotland, remaining active in Aberdeen. He never tired of telling his colleagues that he was one of the few academics north of the border who still led an adult community mental health team. He was also keen to remind them that this team worked within the most deprived catchment area in the city of Aberdeen. As an indication of the great respect with which he was held, his multidisciplinary team readily accepted tertiary referrals of patients with treatment-resistant affective disorder, despite the heavy workload.

Ian Reid was born and brought up in Dunfermline in Fife. His father John was a school head teacher and mother Rae a special education teacher. He was educated at Dollar Academy where he was a brilliant student, gaining entry at the age of 16 to the School of Medicine and Dentistry, University of Aberdeen. After qualifying in medicine, he began his postgraduate training in psychiatry and early academic career in Grampian, with Professor George Ashcroft, before pursuing his higher clinical and academic training, including his PhD in cognitive neuroscience, in Edinburgh, with Professor Richard Morris. After returning to Aberdeen as a clinical senior lecturer, he moved to the University of Dundee as a new Chair of Psychiatry in 1995 at the very young age of 34. In Dundee, Ian became a major force for clinical service change and was involved in establishing community mental health teams and developing a state-of-the-art ECT service. Along with Professor Keith Matthews he established an innovative affective disorders service that provided a specialist clinical service as well as multidisciplinary training and research opportunities for junior psychiatrists, psychologists and nurses. In 2003 he succumbed to the lure of a return to Aberdeen to take up the Chair of Mental Health, a much-cherished post that he held until the time of his death.

Despite his sometimes crusty demeanour, Ian was an incredibly caring, supportive and knowledgeable colleague whose passion for patient care was almost unequalled. His commitment to teaching, training and mentorship was reflected in his ability to persuade non-psychiatric trainees to change their career path to psychiatry. Although at times he could be challenging to trainees and others, he was always driven by a core desire to do all he could to improve patient care and management of those with major mental illness. One of Ian's greatest personal strengths was an ability to connect with anyone at their level. A memory shared by almost all who met Ian would be of his black baggy jumper, well-worn leather jacket and omnipresent can of Coke Zero.

At a recent dedication ceremony at Royal Cornhill Hospital, during which the ECT suite was renamed the ‘Professor Ian C. Reid Centre' in Ian's honour, one of his former patients described Ian as being ‘the best man I ever knew’, a sentiment shared by many who knew him.

Ian is survived by his daughter Alis, of whom he was incredibly proud, his second wife Linda Treliving, a highly respected psychotherapist, his two stepsons, Lawrence and Matthew and his sister Susan, a consultant radiologist.

